# Machine learning-based transcriptmics analysis reveals *BMX*, *GRB10*, and *GADD45A* as crucial biomarkers and therapeutic targets in sepsis

**DOI:** 10.3389/fphar.2025.1576467

**Published:** 2025-03-31

**Authors:** Yanwei Cheng, Haoran Peng, Qiao Chen, Lijun Xu, Lijie Qin

**Affiliations:** ^1^ Department of Emergency, Henan Provincial People’s Hospital, People’s Hospital of Zhengzhou University, People’s Hospital of Henan University, Zhengzhou, China; ^2^ Department of Neurology, People’s Hospital of Henan University, Henan Provincial People’s Hospital, Zhengzhou, Henan, China; ^3^ Nursing Department, Air Force Medical Center, PLA, Beijing, China

**Keywords:** sepsis, biomarkers, transcriptomics, machine learning, therapeutic targets, immune regulation

## Abstract

Sepsis is a life-threatening condition characterized by a dysregulated host response to infection, resulting in high mortality rates and complex clinical management. This study leverages transcriptomics and machine learning (ML) to identify critical biomarkers and therapeutic targets in sepsis. Analyzing microarray data from the Gene Expression Omnibus (GEO) datasets GSE28750, GSE26440, GSE13205, and GSE9960, we discovered three pivotal biomarkers that *BMX* (bone marrow tyrosine kinase gene on chromosome X), *GRB10 (*growth factor receptor bound protein 10), and *GADD45A* (growth arrest and DNA damage inducible alpha), exhibiting exceptional diagnostic accuracy (AUC >0.9). Functional enrichment analyses revealed that these genes play key roles in reactive oxygen species metabolism and immune response regulation. Specifically, *GADD45A* was positively correlated with eosinophils and inversely associated with activated NK cells, CD8 T cells, and activated memory CD4 T cells. *BMX* showed positive correlations with eosinophils, mast cells, and neutrophils, while *GRB10* was linked to eosinophils and M2 macrophages. Additionally, we constructed a comprehensive mRNA-miRNA-lncRNA regulatory network, identifying key interactions that may drive sepsis pathogenesis. Molecular docking and dynamics simulations validated Bendroflumethiazide, Cianidanol, and Hexamidine as promising therapeutic agents targeting these biomarkers. In conclusion, this integrated approach provides profound insights into the molecular mechanisms underlying sepsis, pinpointing *BMX*, *GRB10*, and *GADD45A* as pivotal biomarkers and therapeutic targets. These findings significantly enhance our understanding of sepsis pathophysiology and lay the groundwork for developing personalized diagnostic and therapeutic strategies aimed at improving patient outcomes.

## Introduction

Sepsis is a severe, life-threatening condition characterized by a dysregulated host response to infection, leading to systemic inflammation, multi-organ dysfunction, and high mortality rates (Laura et al., 2021). Globally, sepsis affects approximately 48.9 million individuals annually and accounts for over 11 million deaths, making it a critical public health concern (Kristina et al., 2020). Beyond its immediate lethality, sepsis survivors often endure long-term functional impairments, underscoring the urgent need for more precise diagnostic and therapeutic strategies (Evangelos et al., 2024).

Although widely used clinical biomarkers such as procalcitonin and C-reactive protein provide some prognostic value, they fail to capture the full complexity and dynamic nature of sepsis (Saxena et al., 2024). The pathophysiology of sepsis involves a delicate balance between hyperinflammatory and immunosuppressive responses, complicating efforts to develop effective treatments (Liu D. et al., 2022). Consequently, there is a pressing need for more comprehensive and specific biomarkers that can enhance diagnostic accuracy, predict clinical outcomes, and guide targeted therapies (Cohen and Banerjee, 2024). Traditional approaches to biomarker discovery typically focus on a limited set of molecular factors and often do not account for the multifaceted biological interactions that drive sepsis progression (Pierrakos et al., 2020).

Recent advancements in transcriptomics have significantly improved our understanding of the molecular mechanisms underlying sepsis. Transcriptomic studies have revealed numerous genes implicated in immune dysregulation and disease progression, offering valuable insights into the complex pathophysiology of sepsis (Liu B. et al., 2022). However, analyzing these high-dimensional datasets and identifying clinically meaningful biomarkers remains a challenge (Mohanty et al., 2023). Machine learning (ML) algorithms provide a powerful solution to this complexity. By leveraging computational techniques capable of handling vast amounts of transcriptomic data, ML methods can identify subtle patterns, complex interactions, and critical features that conventional statistical approaches may overlook (Ke et al., 2023). This approach is particularly novel and promising in the context of sepsis, as it enables the comprehensive analysis of thousands of molecular factors and their relationships to immune infiltration patterns. Identifying key transcriptomic biomarkers associated with immune cell dynamics and sepsis outcomes can illuminate disease mechanisms and reveal new therapeutic targets (You et al., 2023). Moreover, ML-driven biomarker discovery has the potential to substantially improve patient risk stratification, inform personalized treatment strategies, and facilitate earlier, more accurate interventions, ultimately improving survival rates and quality of life for sepsis patients.

This study leverages advancements in transcriptomics and ML methodologies to uncover biomarkers and therapeutic targets that can improve sepsis diagnosis and treatment. By combining differential gene expression analysis, weighted gene co-expression network analysis, ML-driven feature selection, functional enrichment analyses, immune cell infiltration profiling, mRNA-miRNA-lncRNA network construction, and *in silico* drug target prediction, we uncover key biomarkers involved in sepsis pathogenesis and explore their therapeutic potential. This integrated approach provides valuable insights into novel therapeutic strategies for sepsis, paving the way for more targeted diagnostic tools and precision therapies in clinical sepsis management.

## Materials and methods

### Data download and preprocessing

High-throughput microarray expression sequencing data for sepsis were retrieved from the Gene Expression Omnibus (GEO) database (https://www.ncbi.nlm.nih.gov/geo/) (Barrett et al., 2013). Four datasets were selected for this study: GSE28750, GSE26440, GSE13205, and GSE9960. The datasets GSE28750, GSE26440, and GSE13205, which include a total of 132 sepsis patients and 60 healthy controls, were used as the training set. These datasets underwent log transformation and batch effect correction using the “Combat” function from the “sva” package in R (Leek et al., 2012). The GSE9960 dataset, consisting of 54 sepsis patients and 16 healthy controls, was designated as the validation set. Detailed information regarding the sample types, group sizes, and inclusion criteria is provided in Supplementary Table S1.

### Screening of potential hub biomarkers in sepsis

Differentially expressed genes (DEGs) between sepsis patients and healthy controls were identified using the “limma” package in R, with statistical thresholds set at |log2 fold change (FC)| > 2 and adjusted P-value (Padj) < 0.05 (Ritchie et al., 2015). The distribution and significance of DEGs were visualized using heatmaps generated by the “pheatmap” and “ggplot2” packages (Ito and Murphy, 2013). To identify gene modules associated with sepsis, we performed weighted gene co-expression network analysis (WGCNA) using the “WGCNA” package in R (Langfelder and Horvath, 2008). All samples were initially clustered to identify and exclude outliers, and genes with similar expression patterns were grouped into modules based on a topological overlap matrix (TOM) derived from the adjacency matrix. The analysis was performed with a deep splitting level of 2, a minimum module size of 100, and a soft-threshold power of 15. Gene significance (GS) and module membership (MM) were calculated for each gene, and modules with a correlation coefficient greater than 0.7 were identified as hub modules for further analysis.

### Screening of hub biomarkers using machine learning

To identify robust biomarkers, 5 ML algorithms were applied to the training datasets. Least Absolute Shrinkage and Selection Operator (LASSO) regression, implemented with the “glmnet” package, was used to shrink regression coefficients and select key features (Waldorp and Haslbeck, 2024). The Random Forest (RF) model, constructed using the “randomForestSRC” package, ranked features based on mean decrease in accuracy (Hu and Szymczak, 2023). Support Vector Machines with Recursive Feature Elimination (SVM-RFE) was performed using the “caret” package, iteratively removing less informative features to optimize prediction accuracy (Sanz et al., 2018). Neural networks were built using the “nnet” package, and Gradient Boosting Machine (GBM) were implemented with the “gbm” package (Salditt et al., 2023). Common features identified across all five methods were visualized using a Venn diagram (Jia et al., 2021). Diagnostic accuracy was assessed using Receiver Operating Characteristic (ROC) curves, with area under the curve (AUC) calculated for each gene.

### Validation of hub biomarkers

The diagnostic performance of the selected biomarkers, *BMX* (bone marrow tyrosine kinase gene on chromosome X), *GRB10* (growth factor receptor bound protein 10), and *GADD45A* (growth arrest and DNA damage inducible alpha), was validated using the GSE9960 dataset. Gene expression levels were visualized with violin plots generated using “ggplot2” in R. ROC curves were generated to evaluate diagnostic accuracy, and AUC values were calculated. Prognostic significance was assessed using cox proportional hazards regression, with hazard ratios (HRs) and 95% confidence intervals visualized in forest plots (Cioci et al., 2021).

### Enrichment analysis and protein-protein interaction network

Gene Ontology (GO), Kyoto Encyclopedia of Genes and Genomes (KEGG), and Disease Ontology (DO) enrichment analyses were performed to understand the functions of genes associated with sepsis, using the “clusterProfiler” and “DOSE” packages in R (Yu et al., 2012). The significance threshold was set at p < 0.05, and the top 15 most significant GO terms and KEGG pathways were visualized with “ggplot2”. Gene Set Enrichment Analysis (GSEA) was used to predict significant biological processes and pathways associated with hub genes, while Gene Set Variation Analysis (GSVA) compared gene set variations between groups, using the “clusterProfiler”, “enrichplot”, and “ggplot2” packages (Kleino et al., 2022). Protein-protein interaction (PPI) networks were constructed using the STRING database (https://cn.string-db.org/) with a confidence score >0.7, and further visualized with Cytoscape (version 3.9.1) (Elisa et al., 2021).

### Immune infiltration analysis

Immune cell infiltration patterns in sepsis and healthy controls were evaluated using the CIBERSORT algorithm, which estimates the relative abundance of 22 immune cell types. Single-sample Gene Set Enrichment Analysis (ssGSEA) was used to score immune-related pathway activities (Kleino et al., 2022). Correlation analyses were conducted to examine the relationships between gene expression levels and immune cell proportions, using Spearman’s rank correlation. The results were visualized as scatter plots, violin plots, and heatmaps.

### Molecular docking and molecular dynamics simulations

Drug-gene interactions were identified using the Comparative Toxicogenomic Database (CTDbase) (https://ctdbase.org/) and Enrichr (https://maayanlab.cloud/Enrichr/). Protein structures were retrieved from UniProt (https://www.uniprot.org/) (Bateman et al., 2022), and molecular docking simulations were performed using AutoDock Vina (version 1.2.0) (Jerome et al., 2021).

A 100-ns molecular dynamics (MD) simulation was conducted using GROMACS 2023 to evaluate the reliability of the protein-drug docking results (Gabriel et al., 2023). The protein structure was parameterized using the CHARMM36 force field, and drug topology was generated with the GAFF2 force field. The protein-drug complex was solvated in a cubic box using the TIP3P water model, and electrostatic interactions were treated with the particle mesh Ewald (PME) method and the Verlet algorithm. Van der Waals and Coulomb interactions were computed with a cutoff of 1.0 nm. The system underwent a 100-ns MD simulation under constant temperature (300 K) and pressure (1 bar) to ensure stability and validate the docking results.

### Statistical analysis

Transcriptome data analysis, ML model construction, and validation were performed using R (version 4.3.3). Molecular docking simulations were conducted using AutoDock Vina (version 1.2.0), and molecular dynamics simulations were performed using GROMACS 2023. Statistical significance was set at p < 0.05.

## Results

### Identification of key candidate genes in sepsis through DEGs and WGCNA

**FIGURE 1:**
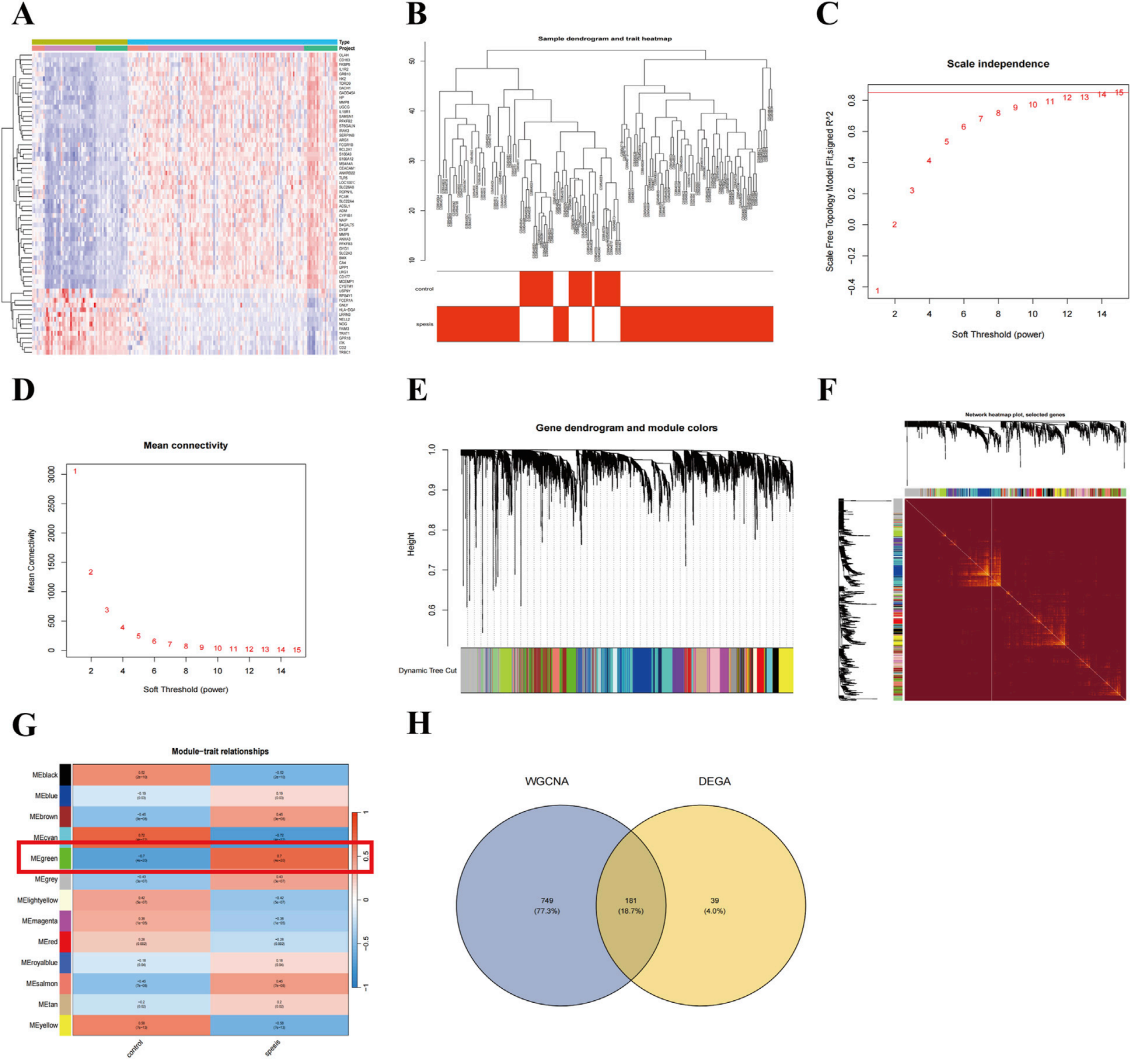
Identification of key candidate genes in sepsis through DEGs and WGCNA. **(A)** Heatmap of differentially expressed genes (DEGs) between sepsis patients and healthy controls. **(B)** Scale-free topology model fit plot for the soft thresholding power. **(C, D)** Mean connectivity for various soft thresholding powers. **(E)** Clustering dendrogram of genes with dissimilarity based on topological overlap, along with assigned module colors. **(F)** The heatmap visualizes the topological overlap matrix (TOM) among the selected genes. **(G)** Correlation between module eigengenes and sepsis status, highlighting the MEgreen module. **(H)** Venn diagram showing the intersection of DEGs and genes in the MEgreen module.

To explore the potential molecular mechanisms of sepsis, we merged and standardized the training datasets, creating an expression matrix with 15,748 genes from 132 sepsis patients and 60 healthy controls (Supplementary Figure S1). Differential expression analysis revealed 175 upregulated and 45 downregulated genes (Figure 1A). A weighted gene co-expression network was constructed with a soft threshold of 15, achieving an R^2^ value of 0.850 (Figures 1B–D). The topological overlap matrix (TOM) was used to perform hierarchical clustering, identifying 13 gene modules, with the MEgreen module (930 genes) showing the strongest positive correlation with sepsis (r = 0.7) (Figures 1E–G). A total of 181 candidate genes were identified from the intersection of DEGs and the MEgreen module (Figure 1H), highlighting their potential role in sepsis pathogenesis.

### Core biomarkers for sepsis identified through ML approaches

**FIGURE 2:**
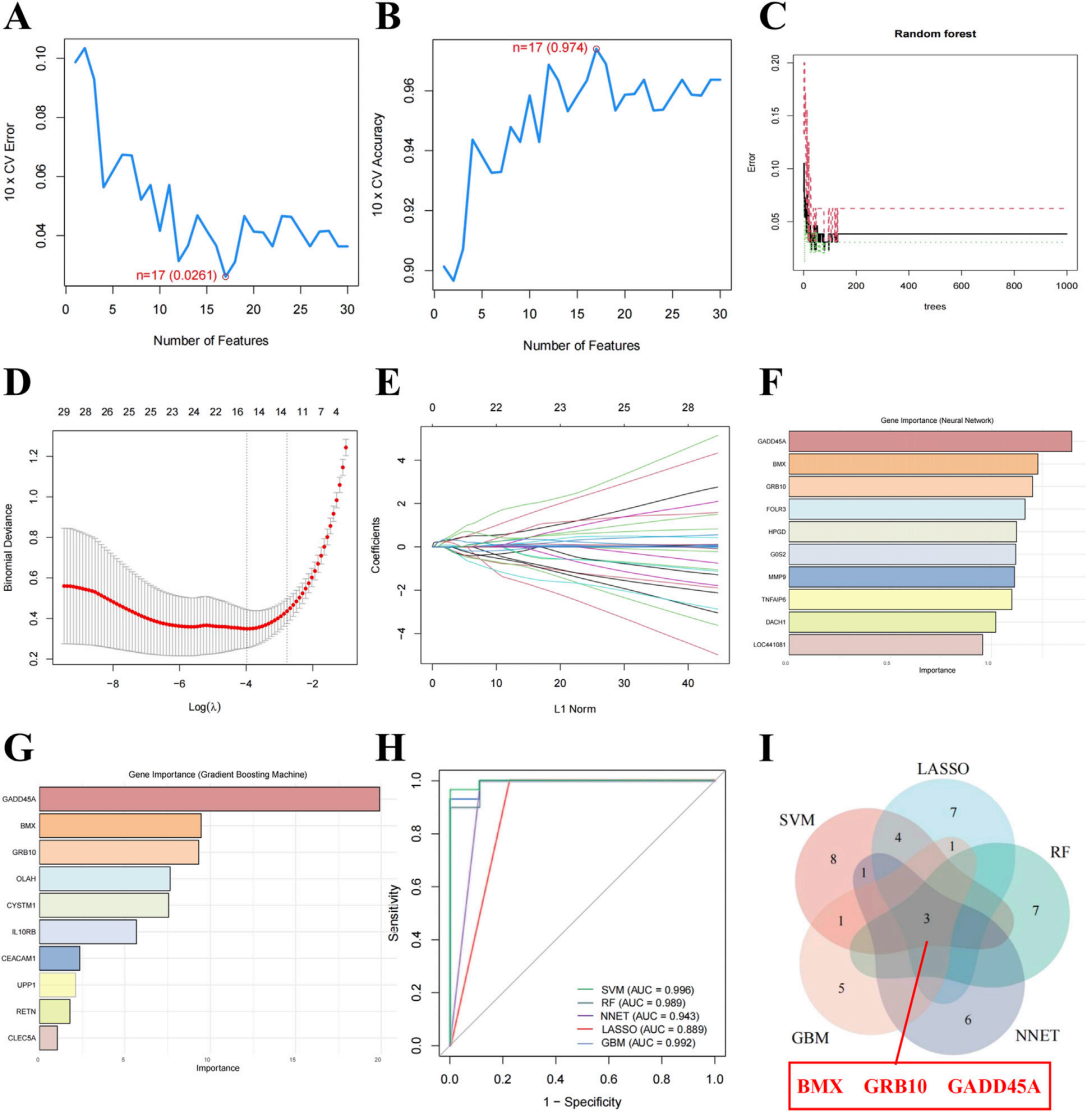
Core biomarkers for sepsis identified through ML approaches. **(A, B)** Root mean square error for Support Vector Machine-Recursive Feature Elimination (SVM-RFE). **(C)** Importance ranking of the top 10 genes identified by Random Forest (RF). **(D)** Coefficient profiles of Least Absolute Shrinkage and Selection Operator (LASSO). **(E)** Optimal lambda selection in LASSO. **(F)** Gene importance scores identified by the Neural Network (NNET) model. **(G)** Gene importance scores identified by the Gradient Boosting Machine (GBM) model. **(H)** Receiver Operating Characteristic (ROC) curves showing the diagnostic accuracy of ML-models. **(I)** Venn diagram highlighting *BMX*, *GRB10*, and *GADD45A* as shared optimal feature genes identified by five ML-models.

To identify key biomarkers with diagnostic potential for sepsis, we applied 5 ML algorithms: SVM-RFE, LASSO, Random Forest, NNET, and GBM (Table 1). SVM-RFE identified 17 genes with the lowest root mean square error (Figures 2A, B), while Random Forest ranked the top 10 genes based on their importance in sepsis-related pathways (Figure 2C). LASSO regression revealed 15 key features at a lambda of 0.040 (Figures 2D, E). NNET and GBM identified 10 genes each, highlighting nonlinear relationships (Figures 2F, G). ROC analysis confirmed the diagnostic potential of all models, with AUC values exceeding 0.7 (Figure 2H). Integration of the outputs from all five algorithms identified *BMX*, *GRB10*, and *GADD45A* as the core biomarkers for sepsis (Figure 2I), reinforcing their reliability for diagnosis.

**TABLE 1:** Key genes outputted by five machine learning algorithms in this study.

Machine learning	Gene Names
LASSO	MCEMP1, S100A9, UPP1, HP, BMX, GADD45A, ANKRD22, ITK, CD2, SLC22A4, NOG, FCER1A, TRAT1, GRB10, HLA-DQA1
RF	BMX, GRB10, GPR84, FAM20A, RAB13, PADI4, S100A12, GADD45A, CPEB4, MMP8
SVM	S100A9, CA4, BMX, TRAT1, GRB10, FCER1A, TLR5, S100P, GADD45A, CYSTM1, CYP1B1, LOC100134822, DACH1, NOG, LRG1, SAMSN1, CD177
NNET	GADD45A, BMX, GRB10, FOLR3, HPGD, G0S2, MMP9, TNFAIP6, DACH1, LOC441081
GBM	GADD45A, BMX, GRB10,OLAH, CYSTM1, IL10RB,CEACAM1, UPP1, RETN, CLEC5A

### Upregulation of *BMX*, *GRB10*, and *GADD45A* in sepsis and their diagnostic performance

**FIGURE 3:**
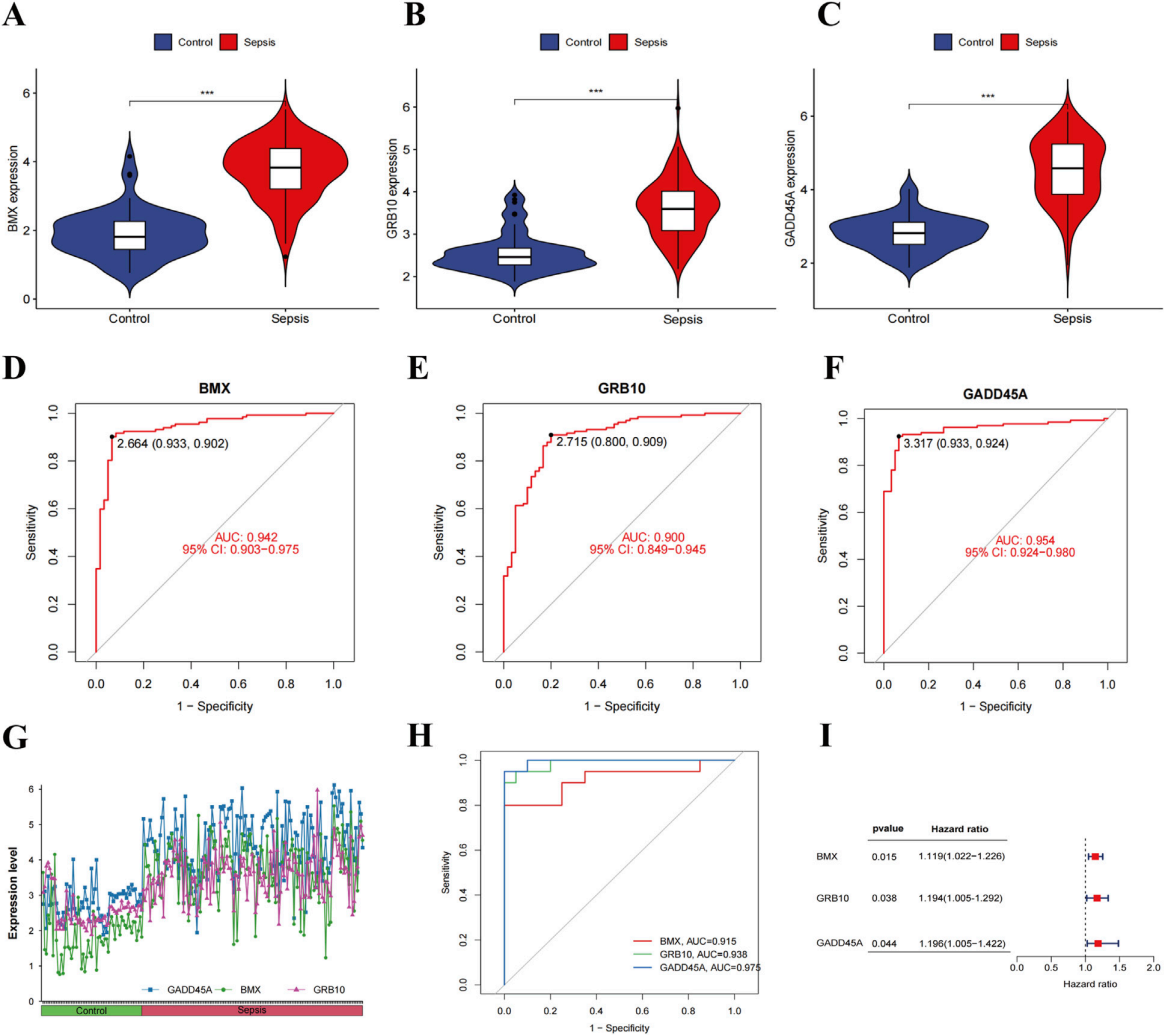
Expression analysis and validation of sepsis-related feature genes. **(A–C)** Violin plots of *BMX*, *GRB10*, and *GADD45A* expression levels in sepsis patients *vs.* healthy controls. **(D–F)** ROC curves for *BMX*, *GRB10*, and *GADD45A* in the training datasets. **(G, H)** Expression levels and ROC curves of *BMX*, *GRB10*, and *GADD45A* in the validation dataset. **(I)** Forest plot of hazard ratios (HR) from cox regression analyses for *BMX*, *GRB10*, and *GADD45A*.

We examined the expression of *BMX*, *GRB10*, and *GADD45A* in sepsis patients, observing significantly elevated expression in sepsis compared to healthy controls (Figures 3A–C). ROC curve analysis demonstrated excellent diagnostic potential, with AUC values of 0.942 for *BMX*, 0.900 for *GRB10*, and 0.954 for *GADD45A* (Figures 3D–F). Validation with the GSE9960 dataset confirmed the upregulation of these genes, with AUCs >0.9 (Figures 3G, H). Cox regression analysis further showed that increased expression of these biomarkers correlated with higher risk of sepsis (HR > 1, Figure 3I), underscoring their diagnostic relevance.

### Functional enrichment and pathway analysis of *BMX*, *GRB10*, and *GADD45A* in sepsis

**FIGURE 4:**
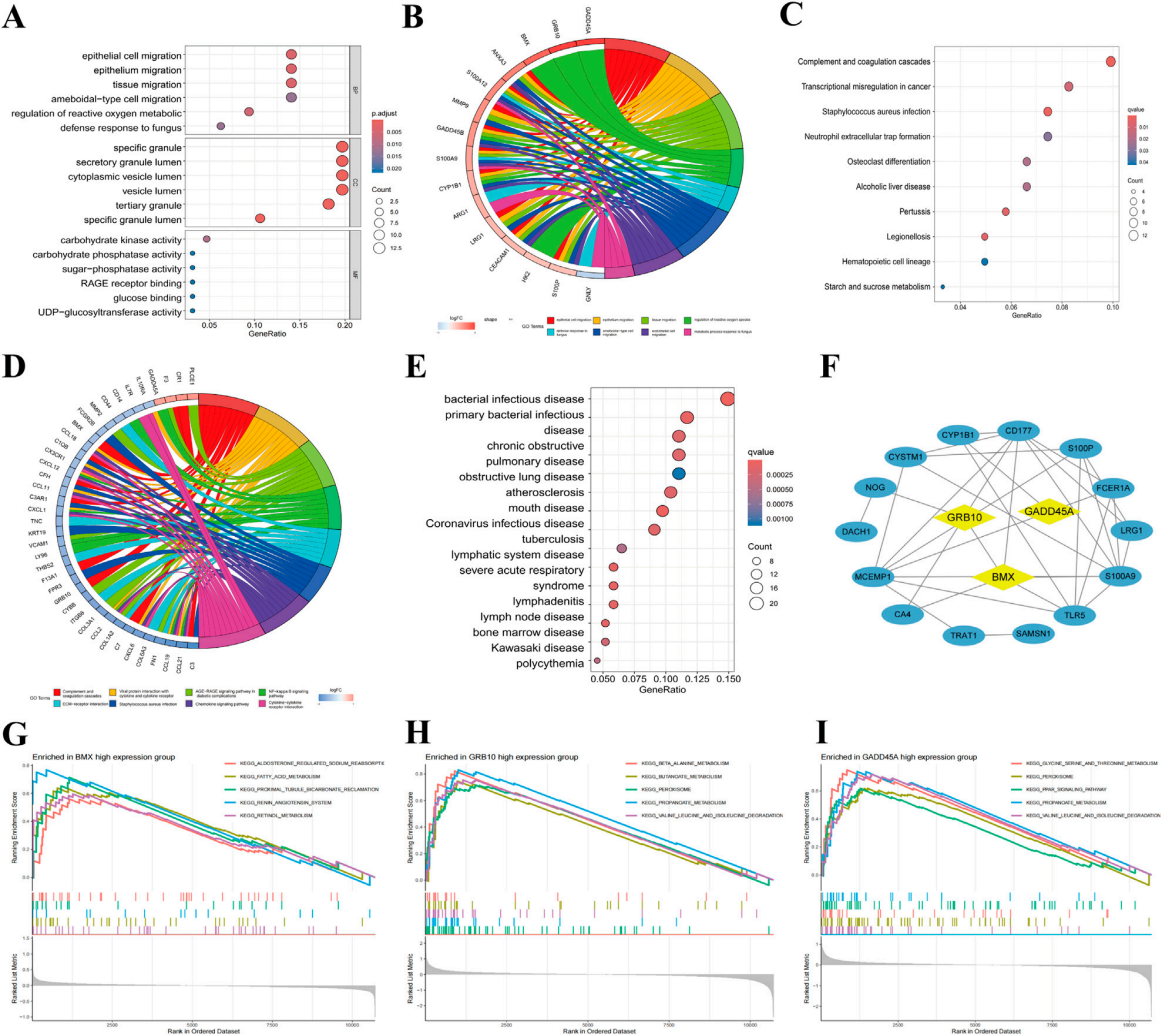
Functional enrichment and pathway analysis of *BMX*, *GRB10*, and *GADD45A* in sepsis. **(A)** Top 15 significant Gene Ontology (GO) terms (Biological Process, Cellular Component, Molecular Function) associated with *BMX*, *GRB10*, and *GADD45A*. **(B)** Chord diagram linking *BMX*, *GRB10*, and *GADD45A* to GO terms. **(C)** Kyoto Encyclopedia of Genes and Genomes (KEGG) pathway analysis highlighting key sepsis-related pathways. **(D)** Chord diagram mapping *BMX*, *GRB10*, and *GADD45A* to their respective KEGG pathways. **(E)** Disease Ontology (DO) analysis indicating bacterial infectious diseases. **(F)** Protein-Protein Interaction (PPI) network of sepsis-related genes. **(G–I)** Gene Set Enrichment Analysis (GSEA) plots for pathways involving *BMX*, *GRB10*, and *GADD45A*.

To explore the biological roles of the identified biomarkers, functional enrichment analyses were performed. GO analysis revealed strong associations with processes such as reactive oxygen species metabolism, cellular stress response, and immune response (Figure 4A). The chord diagram highlighted *BMX*, *GRB10*, and *GADD45A*’s involvement in reactive oxygen species metabolism, a critical pathway in sepsis (Figure 4B). KEGG pathway analysis identified pathways related to complement activation, *staphylococcus aureus* infection, and neutrophil extracellular trap formation (Figure 4C). Among the genes, *BMX* and *GRB10* are particularly associated with immune-regulating signaling pathways, while *GADD45A* is mainly involved in the defense response to bacterial infections (Figure 4D). DO analysis further linked these genes to bacterial diseases such as tuberculosis (Figure 4E). PPI network analysis revealed interactions with key proteins like CD177, S100P, and S100A9, suggesting their roles in immune cell activation and inflammation (Figure 4F). GSEA showed that *BMX*, *GRB10*, and *GADD45A* are involved in several upregulated pathways, such as inflammatory response for *BMX*, cytokine signaling for *GRB10*, and regulation of apoptosis for *GADD45A* (Figures 4G–I). These findings demonstrate that *BMX*, *GRB10*, and *GADD45A* play central roles in the immune and inflammatory mechanisms of sepsis, offering insights into its pathogenesis and potential avenues for intervention.

### Prediction of miRNA and lncRNA regulatory networks for *BMX*, *GRB10*, and *GADD45A*

**FIGURE 5:**
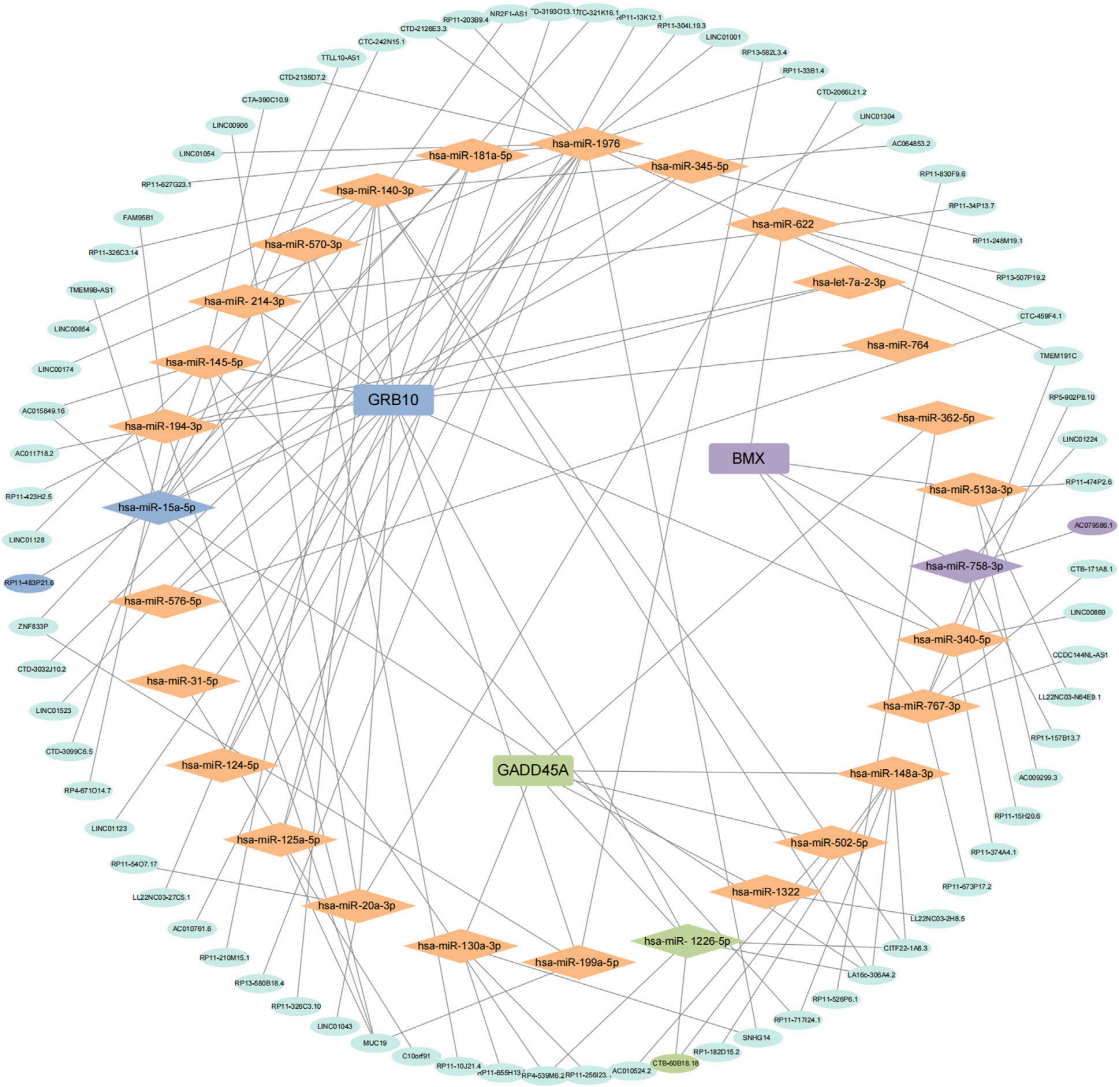
Prediction of miRNA and lncRNA regulatory networks. Competing endogenous RNA (ceRNA) network for *BMX*, *GRB10*, and *GADD45A* showing significant miRNA and lncRNA interactions.

We applied the ceRNA hypothesis to predict the interactions between miRNAs and lncRNAs for the three biomarkers (*BMX*, *GADD45A*, and *GRB10*) (Figure 5). *BMX* was found to interact with 5 miRNAs and 16 lncRNAs, with the *BMX*-miR-758-3p-AC079586.1 pathway showing the highest connectivity. *GADD45A* interacted with 6 miRNAs and 12 lncRNAs, with the *GADD45A*-miR-1226-5p-CTB-60B18.18 pathway showing significant correlation. *GRB10* exhibited the most extensive network, with 53 interactions, and the *GRB10*-miR-15a-5p-RP11-483P21.6 pathway demonstrated the highest correlation. These findings underscore the regulatory roles of *BMX*, *GADD45A*, and *GRB10* in sepsis via complex interactions with miRNAs and lncRNAs.

### Immune microenvironment alterations in sepsis and correlation with hub gene expression

**FIGURE 6:**
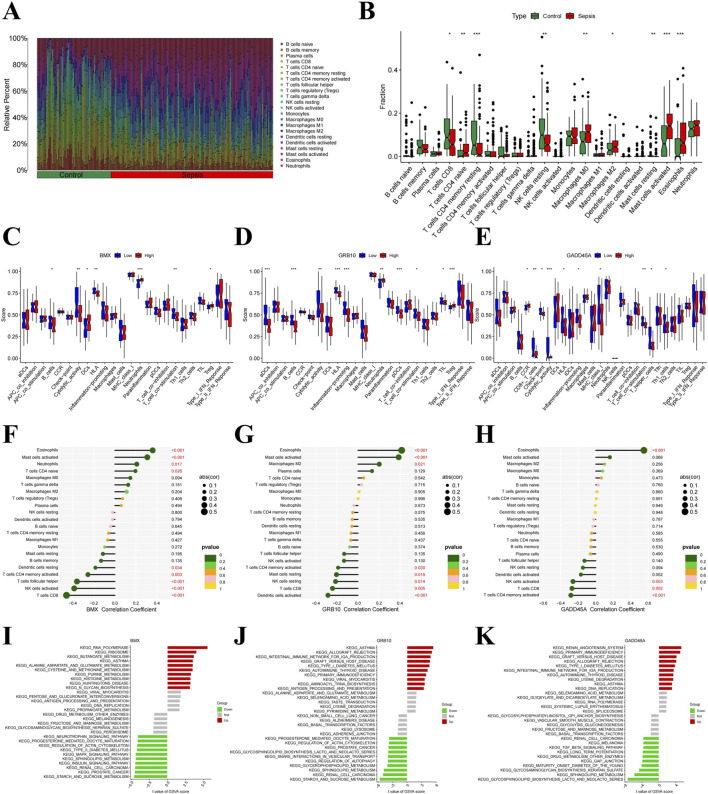
Immune microenvironment alterations in sepsis. **(A)** Barplot of immune cell type proportions in sepsis and healthy samples. **(B)** Violin plot showing significant differences in immune cell infiltration between sepsis and healthy samples. **(C–H)** Correlation plots of *BMX*, *GRB10*, and *GADD45A* with various immune cell types. **(I–K)** Gene Set Variation Analysis (GSVA) of immune pathways involving *BMX*, *GRB10*, and *GADD45A*.

Previous studies have shown that pathogenic genes can alter the immune microenvironment of sepsis (Nicole and Hongbo, 2022). Using the CIBERSORT algorithm, we estimated the abundance of 22 immune cell types and observed significant alterations in immune cell infiltration in sepsis (Figure 6A). Sepsis was associated with increased infiltration of naive CD4 T cells, M0 and M2 macrophages, and activated mast cells, while a decrease was seen in CD8 T cells, resting memory CD4 T cells, and eosinophils (Figure 6B). Further analysis revealed that *BMX* was positively correlated with eosinophils, activated mast cells, and naive CD4 T cells, but negatively correlated with resting dendritic cells and activated NK cells (Figures 6C, F; Supplementary Figure S2A). *GRB10* exhibited positive correlations with eosinophils and M2 macrophages, and negative correlations with follicular helper T cells and activated NK cells (Figures 6D, G; Supplementary Figure S2B). Similarly, *GADD45A* showed positive correlations with eosinophils and negative correlations with activated NK cells and CD8 T cells (Figures 6E, H; Supplementary Figure S2C). GSVA analysis further confirmed the roles of *BMX*, *GRB10*, and *GADD45A* in immune dysregulation during sepsis (Figure 6I), highlighting their involvement in immune environment alterations.

### Drug target prediction for *BMX*, *GRB10*, and *GADD45A* in sepsis treatment

**FIGURE 7:**
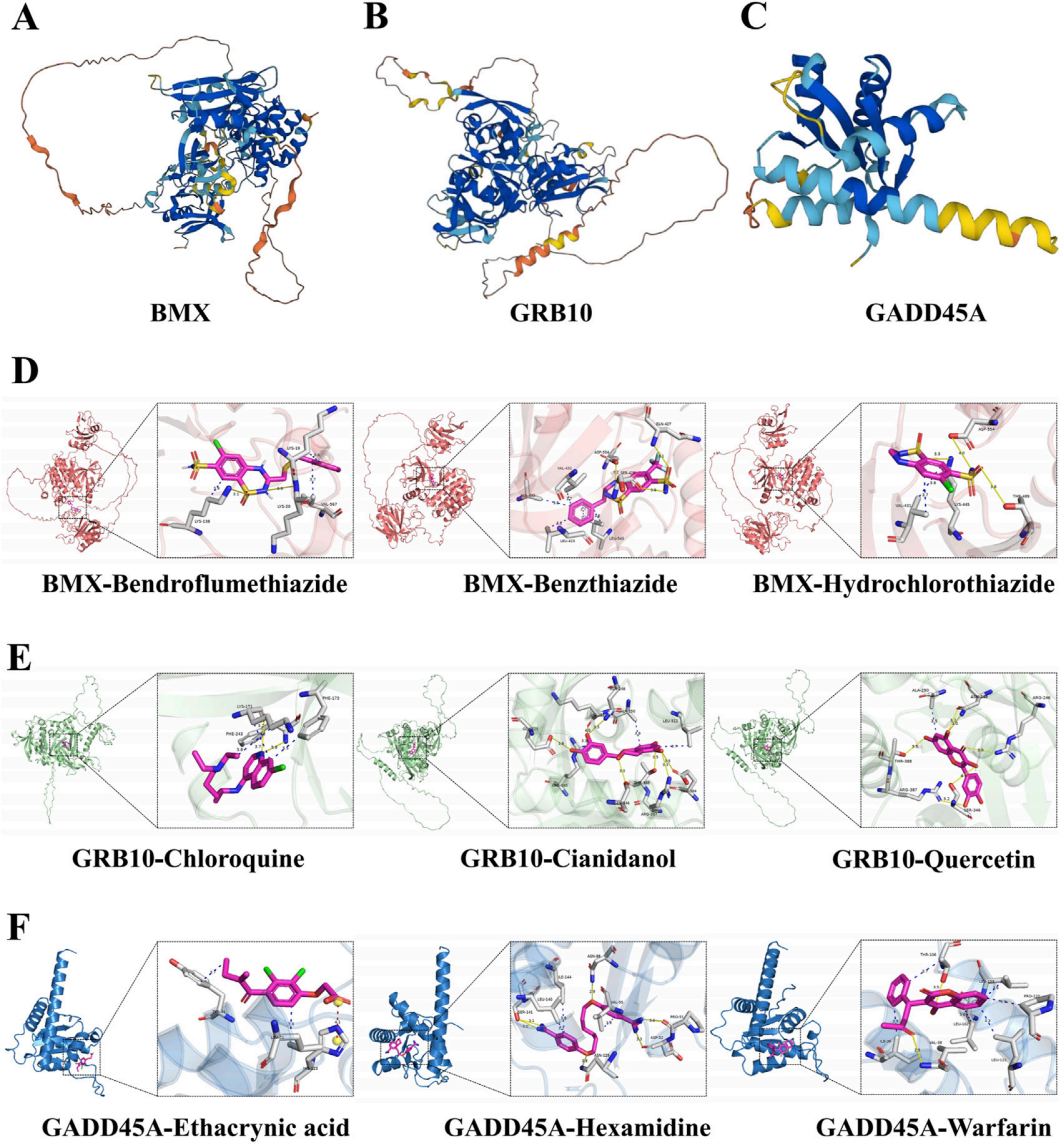
Protein structures and molecular drug docking for *BMX*, *GRB10*, and *GADD45A*. **(A–C)** The protein structures of BMX, GRB10 and GADD45A. **(D–F)** Protein target-small molecule drug docking models showing interactions and binding sites.

To identify effective therapeutic molecules targeting the hub biomarkers *BMX*, *GRB10*, and *GADD45A*, we retrieved protein structures from the UniProt database and selected full-length models from AlphaFold predictions (BMX-HUMAN, GRB10-HUMAN, and GADD45A-HUMAN; Figures 7A–C). We then conducted virtual screening of 2115 FDA-approved small molecules from the ZINC database, selecting the top 10 drugs for each target based on their combined binding scores (Table 2). Molecular docking simulations revealed that Hydrochlorothiazide, Bendroflumethiazide, and Benzthiazide are strong binders for BMX; Chloroquine, Cianidanol, and Quercetin for GRB10; and Warfarin, Hexamidine, and Ethacrynic Acid for GADD45A (Figures 7D–F). Detailed binding interactions, including energy values, bond lengths, and hydrogen bond formations, are summarized in Table 3.

**TABLE 2:** Prediction of target drugs for hub genes.

Gene	IDG drug targets	P-value	Odds ratio	Combined score
BMX	Hydrochlorothiazide	0.006290571	135.3537415	1125.317013
	Bendroflumethiazide	0.006290571	135.3537415	1125.317013
	Benzthiazide	0.006290571	135.3537415	1125.317013
	Phenol	0.00760878	81.20408163	615.4043164
	Ditiocarb	0.00760878	50.74489796	346.3917718
	Salicylic Acid	0.00760878	45.10430839	298.9843051
	Hydroquinone	0.00760878	45.10430839	298.9843051
	Brinzolamide	0.00760878	45.10430839	298.9843051
	Mafenide	0.00760878	45.10430839	298.9843051
	Methazolamide	0.00760878	40.59183673	261.8039189
GRB10	Chloroquine	0.010888425	14.27561328	64.52655689
	Cianidanol	0.015214448	11.75460487	49.1990133
	Quercetin	0.031675223	3.493345164	12.05979787
GADD45A	Warfarin	0.024753529	50.24242424	185.836037
	Hexamidine	0.024753529	50.24242424	185.836037
	Ethacrynic Acid	0.029630963	40.19191919	141.4327674
	Dicoumarol	0.034484249	33.49158249	112.7746184
	Ximelagatran	0.039313504	28.70562771	92.89678501
	Ixabepilone	0.044118846	25.11616162	78.38423124
	Vinflunine	0.044118846	25.11616162	78.38423124
	Carfilzomib	0.044118846	25.11616162	78.38423124
	Cabazitaxel	0.044118846	25.11616162	78.38423124
	Eribulin	0.044118846	25.11616162	78.38423124

**TABLE 3:** Details of molecular docking analysis in this study.

	Binding energy (Kcal/mol)	Hydrogen bonds	Hydrophobic interaction	Salt bridges
BMX-Bendroflumethiazide	−8.3	Ser 425A (3.85 Å), Gln 427A (3.14 Å), Asp 554A (3.19 Å)	Leu 423A (3.80 Å), Val 431A (3.67 Å), Ala 443A (3.63 Å), Leu 543A (3.66 Å, 3.65 Å)	
BMX-Benzthiazide	−7.1	Lys 20A (3.89 Å), Lys 138A (3.15 Å)	Lys 19A (3.64 Å), Lys 138A (3.86 Å), Val 567A (3.70 Å)	
BMX-Hydrochlorothiazide	−6.9	Lys 445A (3.29 Å), Thr 489A (3.78 Å), Asp 554A (4.03 Å)	Val 431A (3.73 Å, 3.84 Å)	
GRB10-Chloroquine	−4.9	Lys 171A (3.15 Å), Phe 243A (3.93 Å)	Lys 171A (3.78 Å, 3.75 Å), Phe 173A (3.48 Å)	
GRB10-Cianidanol	−8.5	Glu 225A (3.90 Å), Asn 248A (3.29 Å), Lys 251A (4.00 Å), Ser 346A (2.98 Å), Glu 384A (3.49 Å), Arg 387A (3.29 Å), Arg 395A (3.30 Å), Thr 388A (3.47 Å, 3.47 Å)	Ala 250A (3.89 Å), Leu 512A (3.75 Å)	
GRB10-Quercetin	−8.5	Arg 246A (3.85 Å), As 387A (3.16 Å), Asn 248A (3.20 Å, 2.99 Å), Ser 346A (3.99 Å, 3.99 Å), Thr 388A (3.52 Å, 3.52 Å)	Ala 250A (3.51 Å)	
GADD45A-Ethacrynic acid	−5.3		Tyr 41A (3.68 Å), Leu 71A (3.61 Å)	His 123A (4.15 Å)
GADD45A-Hexamidine	−5.6	Pro 51A (2.99 Å), Asp 52A (3.25 Å), Asn 88A (2.89 Å), Asn 129A (3.78 Å), Ser 141A (3.10 Å, 3.01 Å)	Val 55A (3.88 Å), Leu 140A (3.25 Å), Ile 144A (3.77 Å)	
GADD45A-Warfarin	−6.5	Val 38A (2.90 Å), Thr 106A (3.45 Å)	Ile 36A (3.46 Å), Leu 102A (3.58 Å), Leu 103A (3.58 Å), Thr 106A (3.71 Å), Pro 120A (3.92 Å), Leu 122A (3.35 Å)	

**FIGURE 8:**
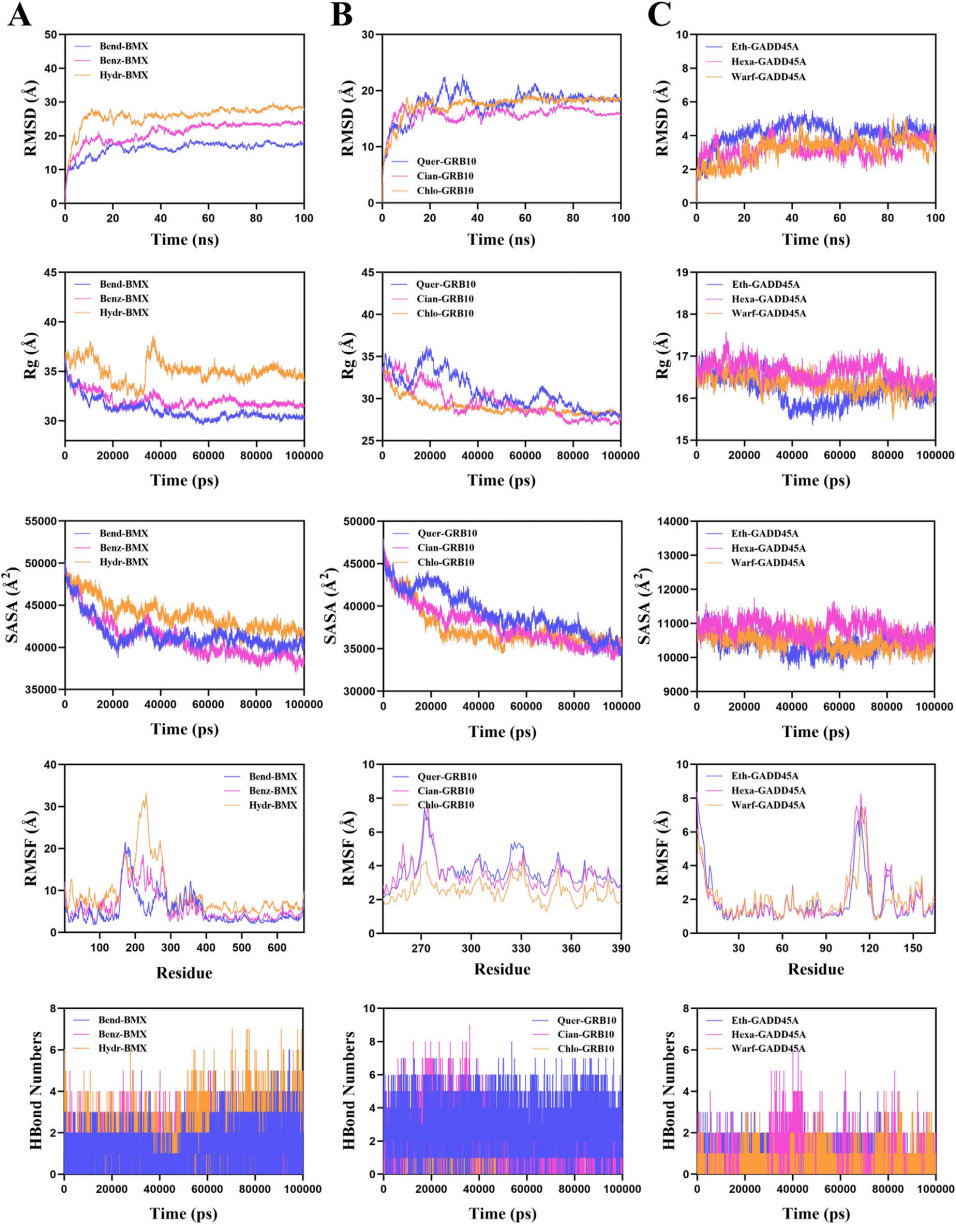
Molecular dynamics simulation results for drug target complexes. Simulation results including Root Mean Square Deviation (RMSD), Radius of Gyration (Rg), Solvent Accessible Surface Area (SASA), Root Mean Square Fluctuation (RMSF), and hydrogen bond numbers for **(A)** Bendroflumethiazide-BMX, Benzthiazide-BMX, and Hydrochlorothiazide-BMX complexes; **(B)** Quercetin-GRB10, Cianidanol-GRB10, and Chlo roquine-GRB10 complexes, and **(C)** Ethacrynic-GADD45A, Hexamidine-GADD45A, and Warfarin-GADD45A complexes.

To confirm the stability of these drug-protein complexes, we performed molecular dynamics simulations. The root mean square deviation (RMSD) values for Bendroflumethiazide-BMX, Cianidanol-GRB10, and Hexamidine-GADD45A were 17.7 Å, 16.4 Å, and 3.6 Å, respectively (Figure 8). Radius of gyration (Rg) and Solvent Accessible Surface Area (SASA) analyses indicated reduced protein flexibility, suggesting stable binding. Additionally, root mean square fluctuation (RMSF) analysis and hydrogen bond data further confirmed strong interactions (Figure 8). These results suggest that Bendroflumethiazide, Cianidanol, and Hexamidine are promising therapeutic candidates for sepsis treatment.

## Discussion

This study utilized an integrated transcriptomics and ML approach to uncover key biomarkers and therapeutic targets in sepsis. The analysis identified *BMX*, *GRB10*, and *GADD45A* as crucial biomarkers with high diagnostic accuracy (AUC >0.9). Functional enrichment and immune cell infiltration analyses highlighted the involvement of these biomarkers in reactive oxygen species metabolism and immune response regulation. Additionally, we constructed a comprehensive mRNA-miRNA-lncRNA regulatory network, identifying critical interactions that may influence sepsis pathogenesis. Docking and molecular dynamics studies further pinpointed potential therapeutic agents, including Bendroflumethiazide, Cianidanol, and Hexamidine, which demonstrated promising binding affinities with these biomarkers.

Previous sepsis research has largely focused on traditional biomarkers such as procalcitonin, C-reactive protein, IL-6, and TNF-α, which are critical mediators of the early inflammatory response. However, their diagnostic utility is constrained by a short detection window and high variability (Pierre et al., 2021). In contrast, our integrative transcriptomics and machine learning approach has identified more specific and robust biomarkers, as demonstrated by the superior diagnostic accuracy of *BMX*, *GRB10*, and *GADD45A*. These biomarkers not only exhibit greater specificity but also provide valuable insights into the immune and metabolic dynamics of sepsis, potentially enhancing their applicability across different disease stages. Although these genes have not been extensively characterized in sepsis, their known functions in other disease contexts provide important clues. *BMX*, a non-receptor tyrosine kinase, participates in inflammatory signaling and can influence vascular integrity (Xiuxiu et al., 2023). *GRB10* modulates insulin signaling and growth factor pathways, thereby affecting cell growth and metabolic homeostasis (Ashlin et al., 2019). *GADD45A* is associated with DNA damage repair, apoptosis, and immune regulation (Mengbing et al., 2024; Markus and KJMRRMR, 2019). These attributes suggest that *BMX* may help regulate endothelial stability and leukocyte trafficking, *GRB10* could shape the metabolic and proliferative states of immune cells, and *GADD45A* might enable immune cells to adapt to prolonged inflammatory stress (Dominic et al., 2012; Deng et al., 2020; She et al., 2023). Collectively, these features position *BMX*, *GRB10*, and *GADD45A* as potential key contributors to the interplay of hyperinflammation, immunosuppression, and oxidative stress that underlies sepsis progression.

Building on these insights, our functional enrichment analyses revealed that *BMX*, *GRB10*, and *GADD45A* are closely linked to critical pathways governing immune responses and reactive oxygen species metabolism. Such pathways are central to sepsis pathogenesis, where a dysregulated immune response and oxidative stress contribute to multi-organ failure (Wang and Liu, 2023). The correlations observed between these biomarkers and specific immune cell subsets further underscore their potential roles in modulating immune cell infiltration, activity, and overall inflammatory balance within the septic milieu. For example, *GADD45A*’s positive correlation with eosinophils and negative correlation with CD8 T cells is consistent with its involvement in calibrating proinflammatory and regulatory immune dynamics, in line with previous evidence of its role in inflammation (Dominic et al., 2012). *BMX’*s positive associations with eosinophils, activated mast cells, and neutrophils align with its capacity to promote inflammatory responses (Deng et al., 2020), while *GRB10’*s correlation with eosinophils and M2 macrophages supports its putative contribution to anti-inflammatory or homeostatic processes (She et al., 2023). These findings highlight the intricate relationships between these biomarkers and immune cell populations, reinforcing the notion that *BMX*, *GRB10*, and *GADD45A* may influence sepsis progression through complex immune regulatory networks.

The construction of the mRNA-miRNA-lncRNA network provides further mechanistic insights. For instance, the *BMX*-miR-758-3p-AC079586.1 and *GRB10*-miR-15a-5p-RP11-483P21.6 axes highlight potential regulatory mechanisms through which non-coding RNAs may influence gene expression and sepsis progression (Tian et al., 2024). Previous studies have demonstrated the critical role of miRNAs and lncRNAs in sepsis by regulating gene expression at the post-transcriptional level. For example, miR-758-3p has been implicated in inflammatory response regulation and cell apoptosis (Peng et al., 2020), while miR-15a-5p has been shown to modulate immune responses and oxidative stress (González-López et al., 2023). The involvement of lncRNAs, such as AC079586.1 and RP11-483P21.6, in sepsis further underscores their potential as therapeutic targets. This network approach underscores the complexity of gene regulation in sepsis and highlights potential targets for therapeutic intervention.

Our docking studies identified several promising therapeutic agents targeting BMX, GRB10, and GADD45A, offering opportunities for drug repurposing and targeted therapy. Specifically, Bendroflumethiazide exhibited strong binding affinity with BMX, Cianidanol showed significant interaction with GRB10, and Hexamidine formed stable complexes with GADD45A. The repositioning of these FDA-approved drugs could accelerate the development of effective sepsis treatments by targeting these newly identified biomarkers.

Despite our robust findings, several limitations must be addressed to fully utilize *BMX*, *GRB10*, and *GADD45A* as sepsis biomarkers and therapeutic targets. Experimental validation is crucial to confirm their roles in sepsis pathogenesis, necessitating cell-based assays with monocytes and endothelial cells using gene overexpression and CRISPR/Cas9-mediated knockdown. Additionally, animal models, such as LPS-induced sepsis and cecal ligation and puncture (CLP) mouse models, will be employed to assess the therapeutic effects of compounds like Bendroflumethiazide, Cianidanol, and Hexamidine on multi-organ damage and inflammation. A further increase in the sample size, along with the support of multicenter studies, is necessary to verify their diagnostic value across diverse populations through qRT-PCR and Western blot analysis of patient samples for clinical validation. Furthermore, experimentally confirming the interactions within the mRNA-miRNA-lncRNA network and integrating additional omics data, such as proteomics and metabolomics, will enhance our understanding of sepsis pathogenesis. These efforts aim to incorporate *BMX*, *GRB10*, and *GADD45A* into diagnostic panels and personalized treatment strategies, thereby improving sepsis management and patient outcomes.

## Conclusion

In conclusion, this study leverages integrated transcriptomics and ML approaches to identify *BMX*, *GRB10*, and *GADD45A* as pivotal biomarkers and therapeutic targets in sepsis. These findings enhance our understanding of sepsis pathophysiology and offer new directions for diagnostic and therapeutic strategies. The identified biomarkers exhibit high diagnostic accuracy and are involved in key pathogenic pathways, providing potential targets for personalized medicine.

## Data Availability

Publicly available datasets were analyzed in this study. These data can be found here: https://www.ncbi.nlm.nih.gov/geo/, using accession numbers GSE28750, GSE26440, GSE13205, and GSE9960.

## Abbreviations

AUC, area under the curve; BMX, bone marrow tyrosine kinase gene on chromosome X; CLP, cecal ligation and puncture; DEGs, differentially expressed genes; DO, Disease Ontology; GADD45A, growth arrest and DNA damage inducible alpha; GEO, Gene Expression Omnibus; GO, Gene Ontology; GRB10, growth factor receptor bound protein 10; GS, Gene significance; GSEA, Gene Set Enrichment Analysis; GSVA, Gene Set Variation Analysis; LASSO, Least Absolute Shrinkage and Selection Operator; MD, molecular dynamics; ML, machine learning; MM, module membership; KEGG, Kyoto Encyclopedia of Genes and Genomes; Rg, Radius of gyration; RF, Random Forest; ROC, Receiver Operating Characteristic; RMSD, root mean square deviation; RMSF, root mean square fluctuation; SASA, solvent accessible surface area; SVM-RFE, Support Vector Machines with Recursive Feature Elimination; ssGSEA, Single-sample Gene Set Enrichment Analysis; TOM, topological overlap matrix; WGCNA, weighted gene co-expression network analysis.
