# Supplementary material for: Machine learning-based transcriptmics analysis reveals BMX, GRB10, and GADD45A as crucial biomarkers and therapeutic targets in sepsis
Source: Front Pharmacol. 2025 Mar 31;16:1576467. doi: 10.3389/fphar.2025.1576467 (PMC11994739; doi:10.3389/fphar.2025.1576467)
Supplement: Supplementary file 1 [file Table1.pdf]

Supplementary table 1. List of datasets and platforms utilized in this study

		Training dataset			Validation dataset
		GSE28750	GSE26440	GSE13205	GSE9960
Platform ID		GPL570	GPL570	GPL570	GPL570
Sample Source		Whole blood	Whole blood	Skeletal muscle	Whole blood
Sample Countries		Australia	America	United Kingdom	Australia
Number of sepsis cases		21	98	13	54
Number of healthy controls		20	32	8	16
Inclusion Criteria	Sepsis group	1. Diagnosed according to the sepsis consensus statement criteria;<br>2. Possess clinical evidence of systemic infection in microbiological diagnosis.	Pediatric patients admitted to ICU.	Sepsis induced multiple organ failure of ICU.	Sepsis from non-infectious causes of systemic inflammatory response syndrome.
	Healthy Control	Hospital staff without known illnesses.	Random healthy individuals.	Age matched healthy controls.	Random healthy individuals.
Data grouping		Sepsis / Sepsis (postoperative) / Healthy controls	Sepsis / Sepsis (septic shock)/Health controls	Sepsis / Healthy controls	Sepsis (Gram-positive) / Sepsis (Gram-negative) / Health controls
