# Supplementary figures and images for: Machine learning-based transcriptmics analysis reveals BMX, GRB10, and GADD45A as crucial biomarkers and therapeutic targets in sepsis
Source: Front Pharmacol. 2025 Mar 31;16:1576467. doi: 10.3389/fphar.2025.1576467 (PMC11994739; doi:10.3389/fphar.2025.1576467)

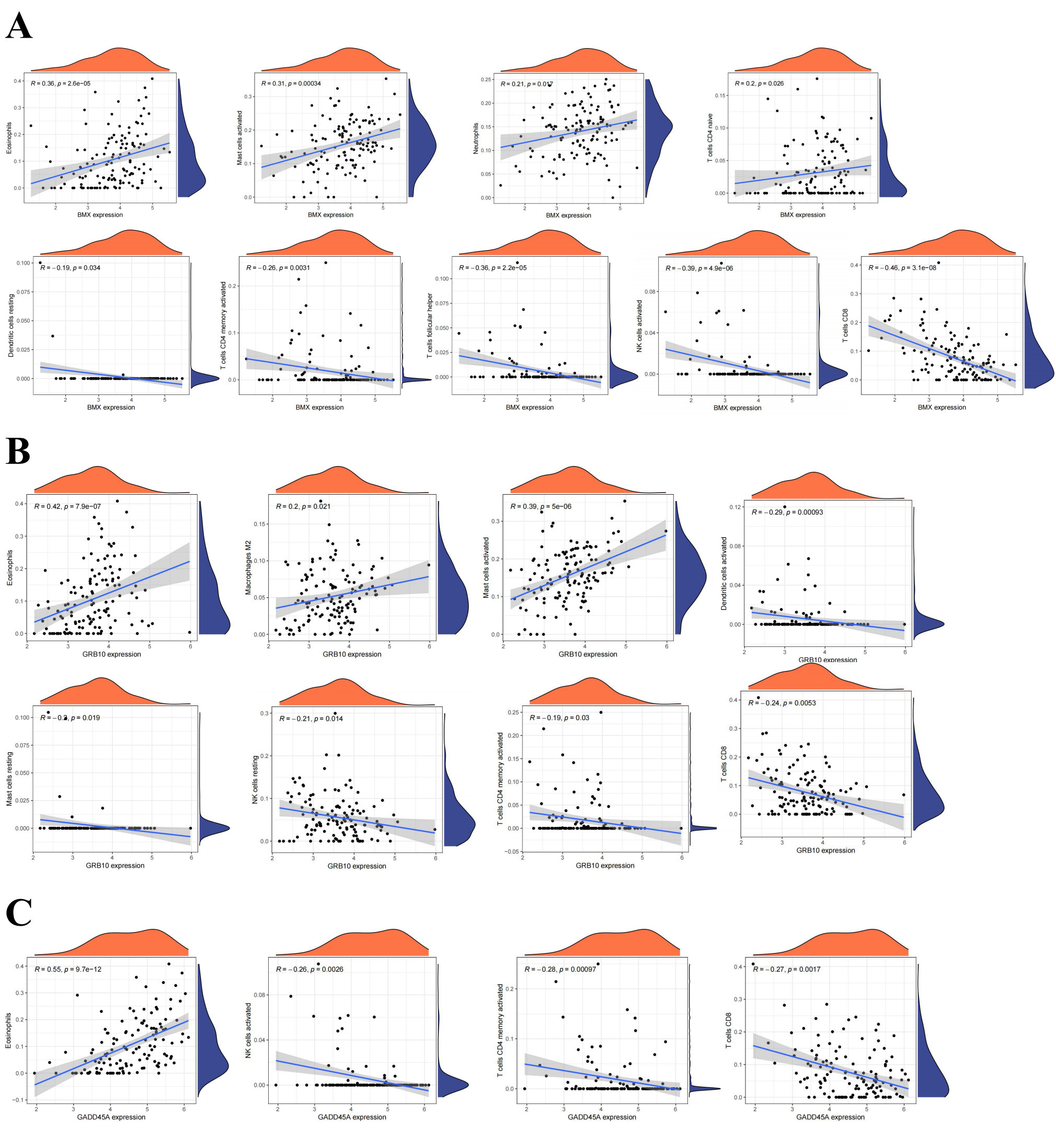

Supplement: Supplementary file 2 [file Image2.tif]

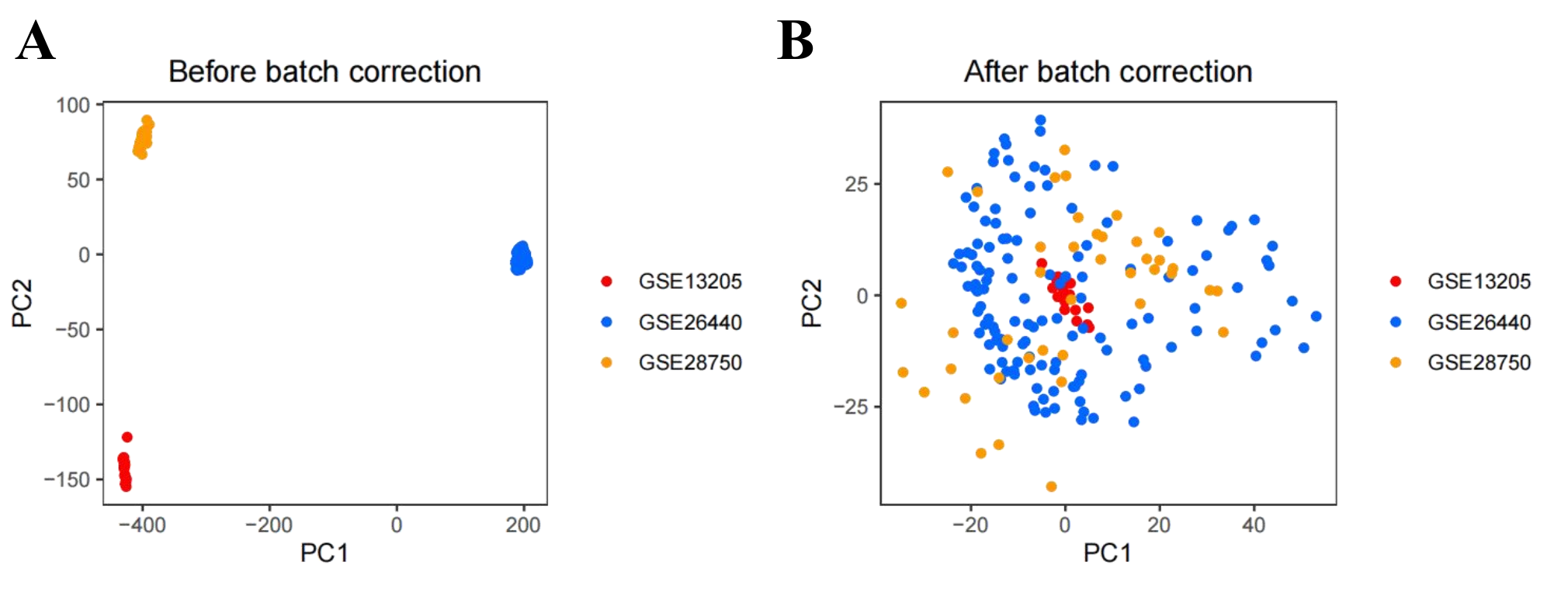

Supplement: Supplementary file 3 [file Image1.tif]
